# Supplementary figures and images for: DeLTA: Automated cell segmentation, tracking, and lineage reconstruction using deep learning
Source: PLoS Comput Biol. 2020 Apr 13;16(4):e1007673. doi: 10.1371/journal.pcbi.1007673 (PMC7153852; doi:10.1371/journal.pcbi.1007673)

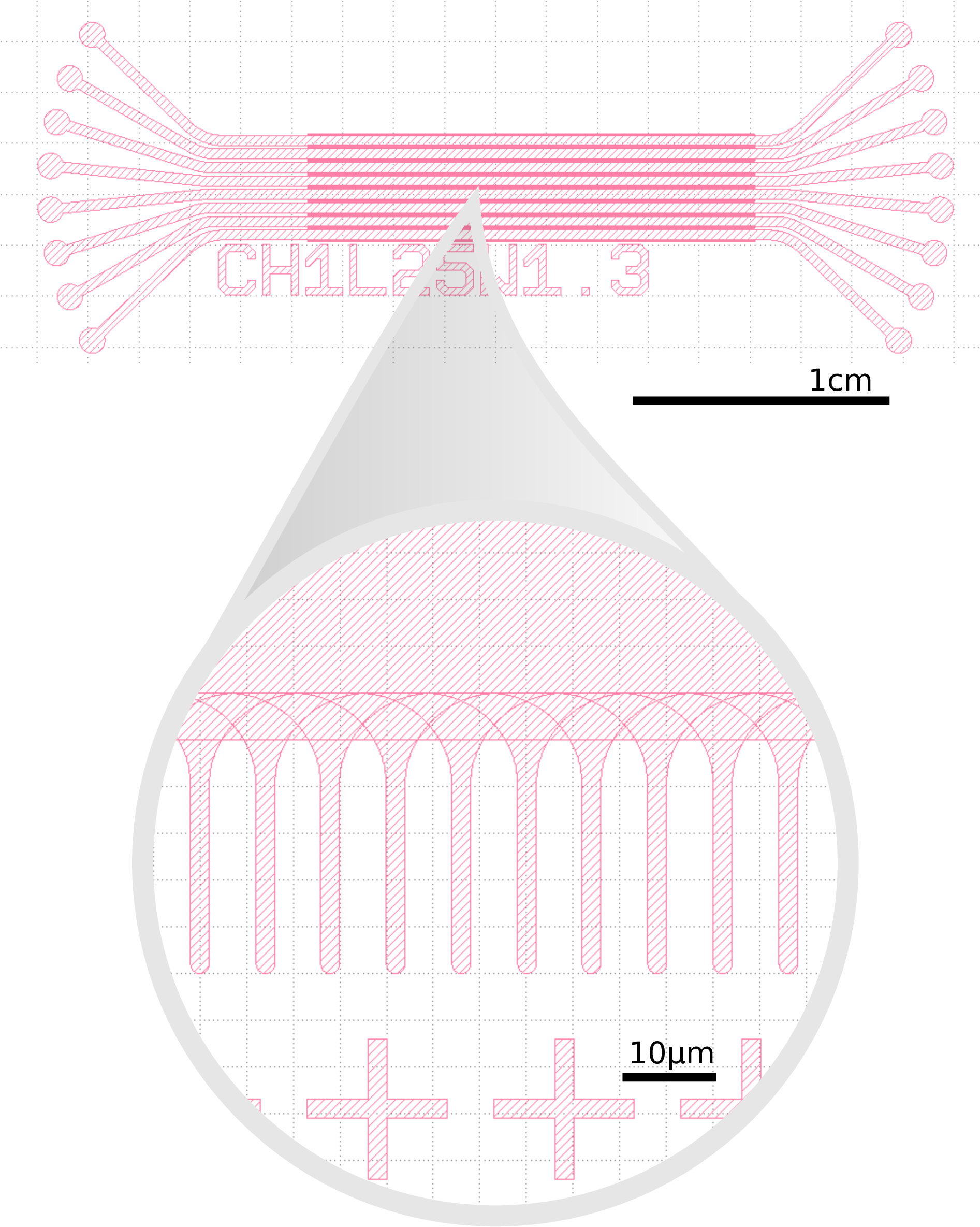

Supplement: S1 Fig — This image is adapted from the mask design file of the mother machine microfluidic chip that was used to generate the training and evaluation data. The upper half of the figure shows the entire chip which features 8 independent main channels of 400μm in width and ~60μm in height in which media containing nutrients and selection markers is flown. Each of those channels features on its side 6,000 chambers of 1.1μm in height, 25 or 35μm in length, and 1.3 to 1.8μm in width in which mother cells are trapped and can then be grown for hours to days. (TIF) [file pcbi.1007673.s001.tif]

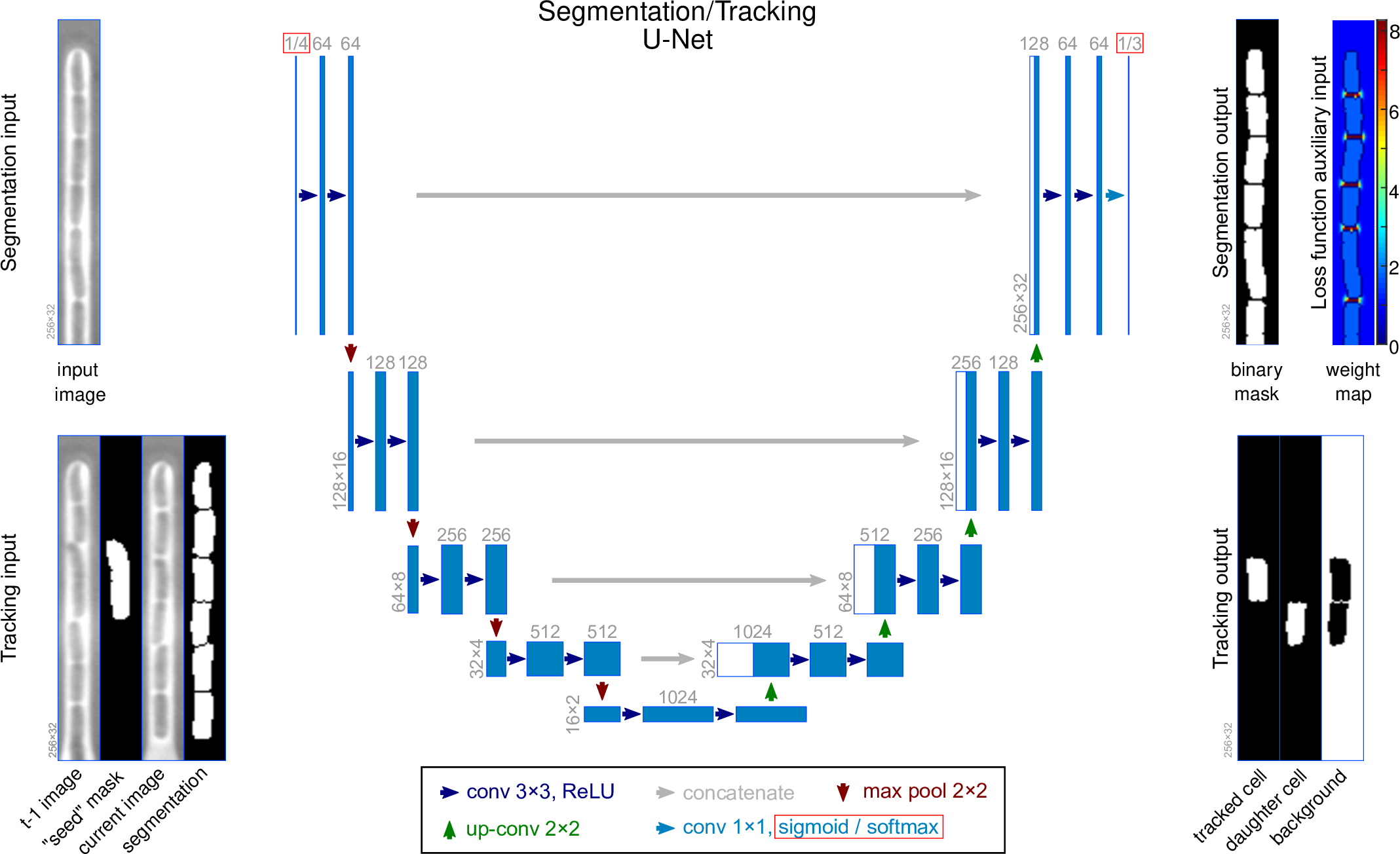

Supplement: S2 Fig — The layers and tensor dimensions used in our U-Net implementation. The differences between the segmentation and the tracking U-Net are highlighted with red rectangular boxes. The first element is for the segmentation U-Net version, the second for the tracking version (segmentation/tracking). Note that the architecture is the same as the original U-Net model, except for image dimensions, number of input and output layers, and the final activation layer for tracking. The values we used for these are noted on the figure. The loss function for segmentation is a pixel-wise weighted binary cross-entropy as described in the original U-Net paper. The pixel-wise weight maps are provided for each output mask during training as an auxiliary input. The loss function for tracking is the standard categorical cross-entropy function from Keras. (TIF) [file pcbi.1007673.s002.tif]

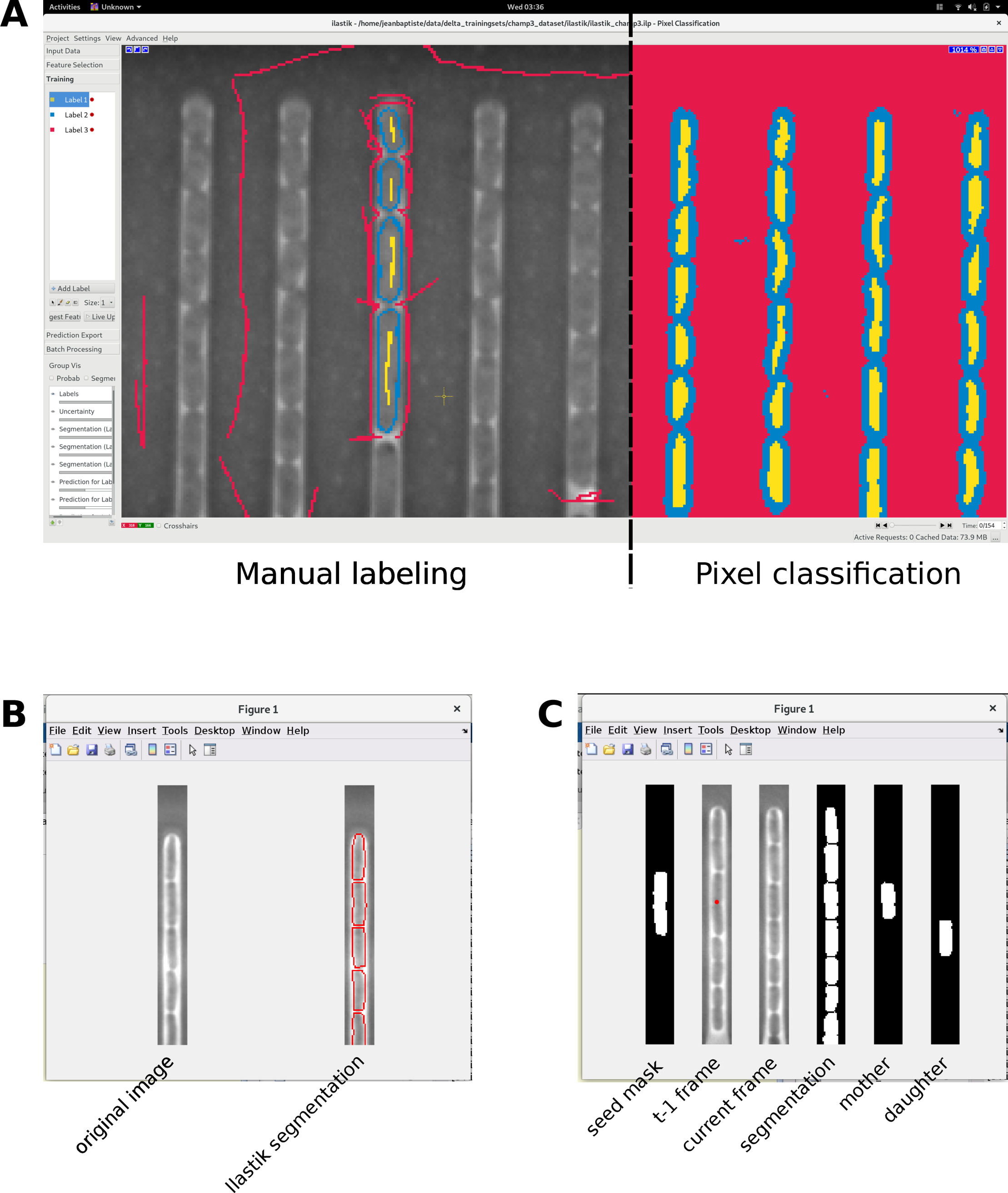

Supplement: S3 Fig — (A) We used the Ilastik software to generate initial segmentation masks for training. The Ilastik software uses a random forest classifier and various local pixel features to classify pixels in an image on the basis of a training set drawn by the user. On the left side of the image are the three classes we use to generate initial segmentation masks and an example of how we draw Ilastik training sample sets: The first label (or class) is dedicated to the internal part of the cells, the second label is used to delineate cell borders, and the third is used for everything else. On the right-hand side is an example of the pixel-wise classification output generated by Ilastik after training on our drawn training set input. While the result is not perfect, this approach allowed us to generate a large number of potential training samples with minimal manual effort. (B) The Ilastik output was then processed via Matlab, where a few simple mathematical morphology operations were used to remove small isolated pixel regions that have been misclassified. We then performed a watershedding operation from the first “internal” label/class into the second “border” class of the Ilastik output. This operation segments separated cell regions in the Ilastik output. The user is then prompted with randomly selected segmentation samples as illustrated in this image. If the user considers the training sample correct, the user can press the “enter” key and the sample is saved to disk as a training sample for the segmentation U-Net. If not, they can press “q” and the sample is saved to disk in a separate folder should the user want to correct it manually. (C) For tracking set generation, the user is presented with a randomly selected sample from the processed Ilastik output, and a cell from the previous timepoint is randomly selected as the “seed” cell to track as in this image. The user can then click on where they think the cell is in either the “current frame” or “segmentation” image in the [file pcbi.1007673.s003.tif]

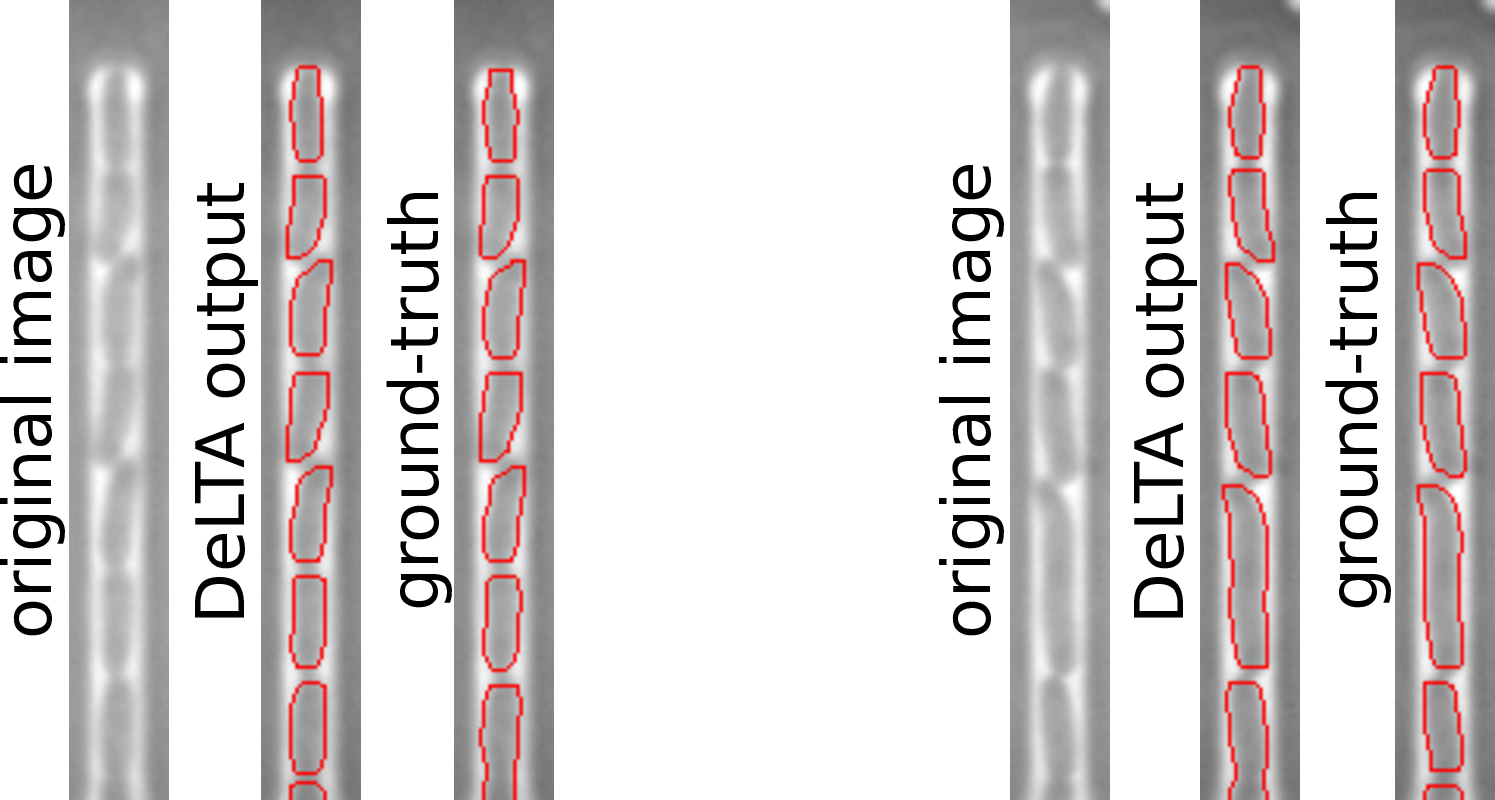

Supplement: S4 Fig — Only 2 segmentation errors out of 3,422 cells were identified when computing the error rate of our trained U-Net against the ground-truth for our evaluation movie. The error on the left illustrates an over-segmentation error, where the bottom cell has been divided by our algorithm when the ground-truth has not. The error on the right illustrates under-segmentation where two cells in the ground-truth have been identified as a single one by DeLTA. For the second error, the DeLTA output could arguably be considered to be correct and the ground-truth erroneous, as the exact point of cell division is sometimes difficult to discern. (TIF) [file pcbi.1007673.s004.tif]

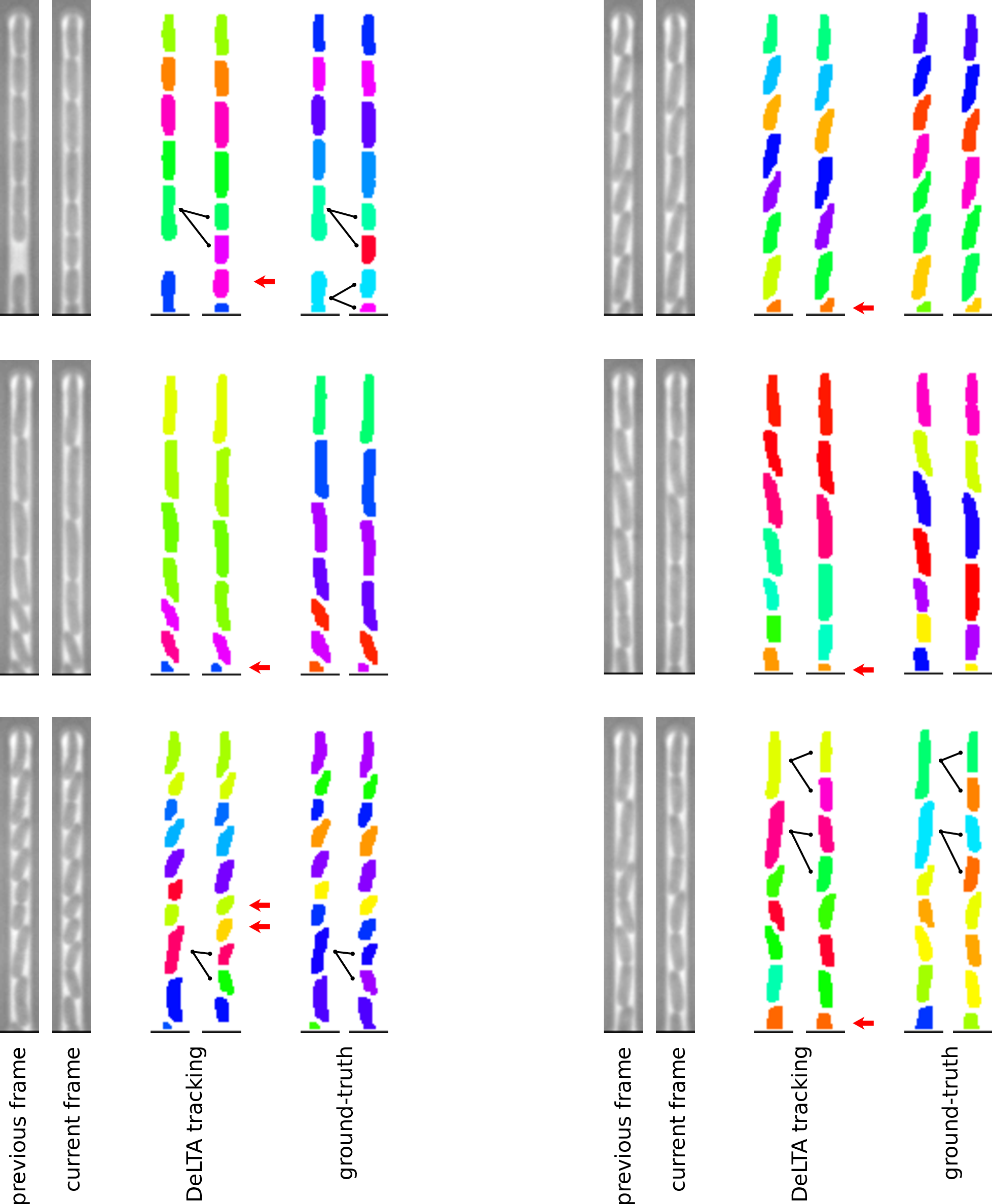

Supplement: S5 Fig — 31 errors were identified out of 3,073 tracking events in the evaluation movie acquired in our lab. Here we show seven representative errors. For each set of images, the raw input images are shown on the left for the current and previous timepoint. The two images in the center illustrate the DeLTA tracking output. Each cell is color coded and the color is preserved from one timepoint to the next. Divisions are identified with black solid lines. Tracking errors are signaled by red arrows. On the right, the ground-truth data are shown for reference. Note that most errors occur as cells are being flushed out of the chambers. (TIF) [file pcbi.1007673.s005.tif]

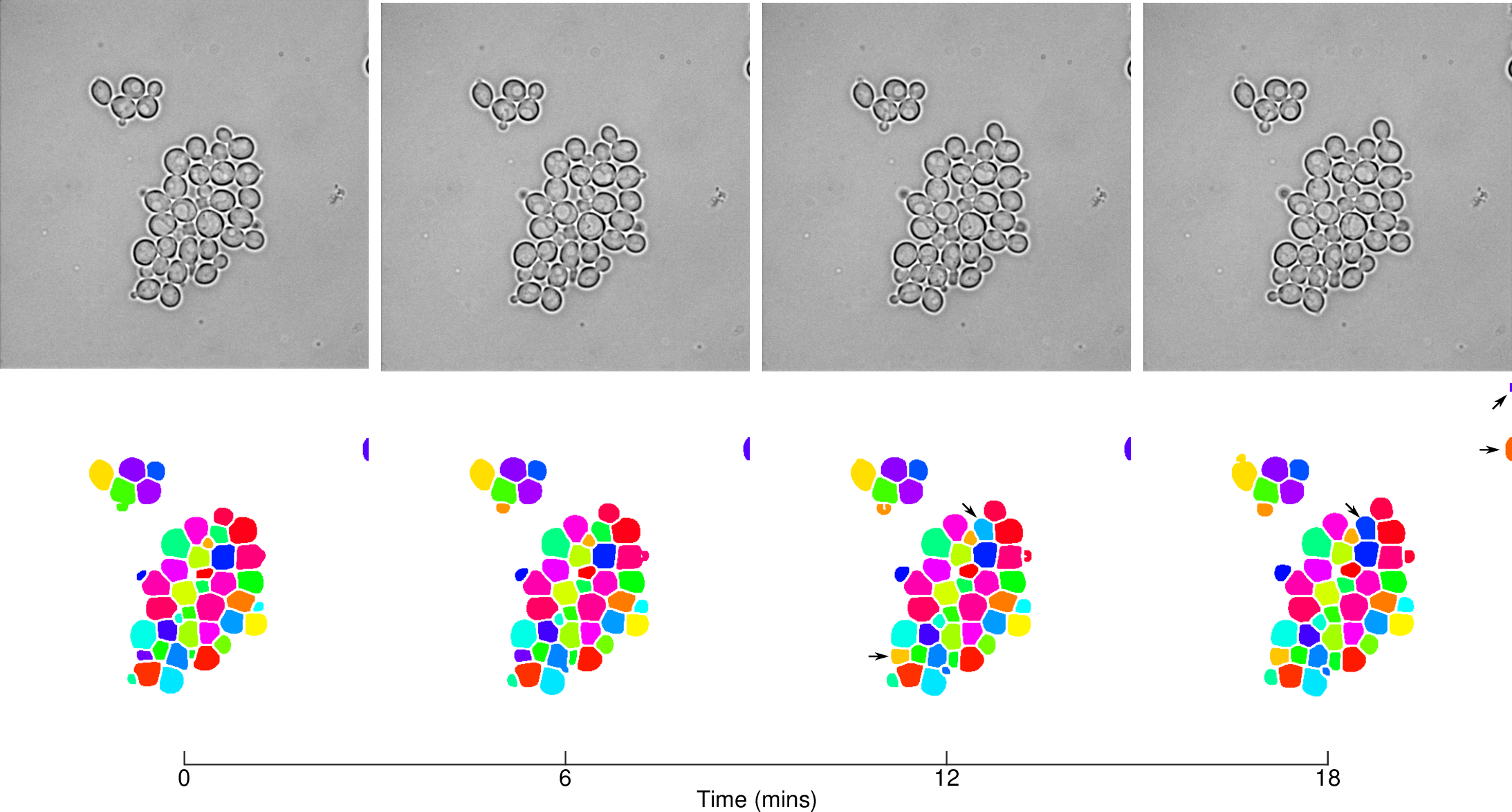

Supplement: S6 Fig — The yeast time-lapse movies datasets are from the Cellstar sample datasets. Yeast cells were imaged with a 60X phase contrast objective in a microfluidic device that constrained them to a monolayer. Black arrows highlight tracking and segmentation errors. Note that the original training sets did not feature mother-daughter relationship information so we did not train the tracking U-Net to identify daughter cells. The two U-Net models were not trained on data from the experiment shown here, but on other experiments in the dataset. While almost all cells are correctly segmented, tracking appears to be more erroneous. However, the datasets that were available online were significantly smaller than the mother machine movies we had (In total there are 170 movie frames in the yeast dataset, with most of them containing only a few cells, when the mother machine movies contain thousands of frames and tens of thousands of cells.) and therefore the training sets may not be diverse enough for DeLTA to be able to generalize tracking rules to new data. We anticipate this would improve with additional training data. (TIF) [file pcbi.1007673.s006.tif]

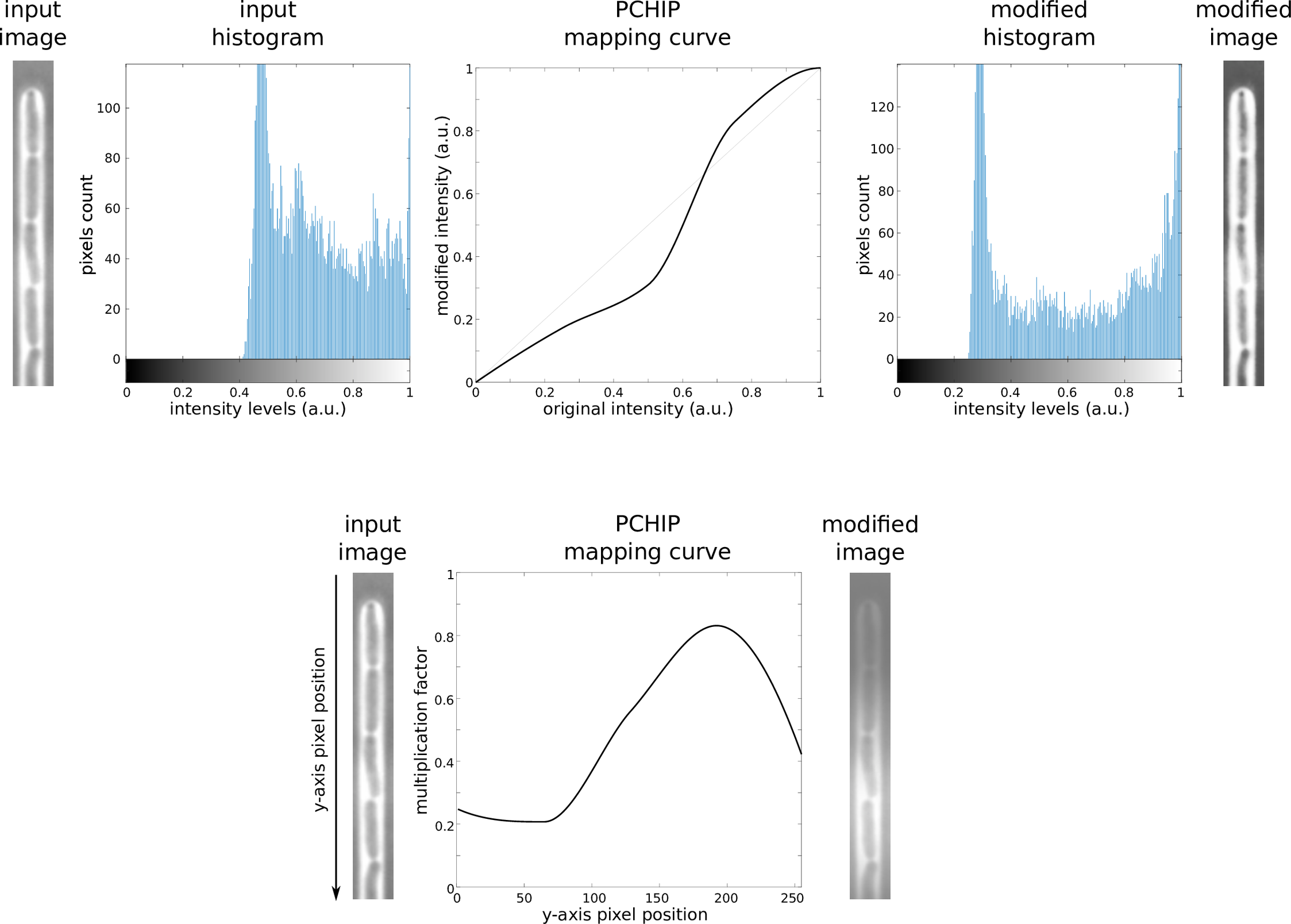

Supplement: S7 Fig — In order to simulate changes in illumination between different experiments or different microscopes, we implemented two data augmentation operations. Two representative examples are given here. The first example of data augmentation (top row) generates a random monotonically increasing Piecewise Cubic Hermite Interpolating Polynomial (PCHIP) curve that is used to distort the intensity histogram of the input image. The second data augmentation example (bottom row) generates another PCHIP curve that is multiplied against image intensity along the y-axis (along the chamber) to simulate imaging artefacts caused by microfluidic features in the phase contrast light path. After multiplication, the image intensity range is mapped linearly back to the original range. (TIF) [file pcbi.1007673.s007.tif]
