# Supplementary material for: DeLTA: Automated cell segmentation, tracking, and lineage reconstruction using deep learning
Source: PLoS Comput Biol. 2020 Apr 13;16(4):e1007673. doi: 10.1371/journal.pcbi.1007673 (PMC7153852; doi:10.1371/journal.pcbi.1007673)
Supplement: S1 Table — (DOCX) [file pcbi.1007673.s008.docx]

| **Leave-one-out Evaluation – Molyso left out** | | |
| --- | --- | --- |
|  | Segmentation | Tracking |
| Ground-truth set size | 1,874 cells | 1,514 cells |
| Errors (rate) | 4 (0.2%) | 46 (3.03%) |
| **Leave-one-out Evaluation – BACMMAN left out** | | |
|  | Segmentation | Tracking |
| Ground-truth set size | 1,785 cells | 1,504 cells |
| Errors (rate) | 4 (0.22%) | 8 (0.53%) |
| **Leave-one-out Evaluation – MoMA left out** | | |
|  | Segmentation | Tracking |
| Ground-truth set size | 1,037 cells | 216 cells |
| Errors (rate) | 179 (17.3%) | 4 (1.85%) |

**S1 Table. Leave-one-out evaluation of the DeLTA algorithm against other mother machine software analysis datasets.**
